# Caveats of Using Overexpression Approaches to Screen Cellular Host IFITM Proteins for Antiviral Activity

**DOI:** 10.3390/pathogens12040519

**Published:** 2023-03-27

**Authors:** Tina Meischel, Svenja Fritzlar, Fernando Villalón-Letelier, Jeffrey M. Smith, Andrew G. Brooks, Patrick C. Reading, Sarah L. Londrigan

**Affiliations:** 1 Department of Microbiology and Immunology, University of Melbourne, The Peter Doherty Institute for Infection and Immunity, 792 Elizabeth St., Melbourne, VIC 3000, Australia; 2 WHO Collaborating Centre for Reference and Research on Influenza, Victorian Infectious Diseases Reference Laboratory, The Peter Doherty Institute for Infection and Immunity, 792 Elizabeth St., Melbourne, VIC 3000, Australia

**Keywords:** respiratory viruses, innate immunity, IFITM proteins, host antiviral factors, influenza A virus, parainfluenza virus

## Abstract

Ectopic protein overexpression in immortalised cell lines is a commonly used method to screen host factors for their antiviral activity against different viruses. However, the question remains as to what extent such artificial protein overexpression recapitulates endogenous protein function. Previously, we used a doxycycline-inducible overexpression system, in conjunction with approaches to modulate the expression of endogenous protein, to demonstrate the antiviral activity of IFITM1, IFITM2, and IFITM3 against influenza A virus (IAV) but not parainfluenza virus-3 (PIV-3) in A549 cells. We now show that constitutive overexpression of the same IFITM constructs in A549 cells led to a significant restriction of PIV-3 infection by all three IFITM proteins. Variable IFITM mRNA and protein expression levels were detected in A549 cells with constitutive versus inducible overexpression of each IFITM. Our findings show that overexpression approaches can lead to levels of IFITM1, IFITM2, and IFITM3 that significantly exceed those achieved through interferon stimulation of endogenous protein. We propose that exceedingly high levels of overexpressed IFITMs may not accurately reflect the true function of endogenous protein, thus contributing to discrepancies when attributing the antiviral activity of individual IFITM proteins against different viruses. Our findings clearly highlight the caveats associated with overexpression approaches used to screen cellular host proteins for antiviral activity.

## 1. Introduction

Innate antiviral signalling in host cells involves the induction of type I and type III interferon (IFN) responses to orchestrate the expression of numerous cellular factors, including interferon stimulated genes (ISGs) that can modulate viral infection and contribute to cellular intrinsic immunity (reviewed in [1,2]). In vitro overexpression approaches have been widely used to investigate host proteins for their ability to mediate antiviral activity. These approaches have proven useful in defining the spectrum of antiviral activity against different viruses, as well as identifying the critical domains of the host protein of interest required for virus inhibition. Seminal work by Brass and colleagues used overexpression approaches to demonstrate the antiviral activity of interferon-inducible transmembrane (IFITM) proteins against influenza A virus (IAV), West Nile virus (WNV), and dengue virus (DENV) [3]. Subsequent studies have utilised IFITM overexpression approaches to show the restriction of a number of respiratory viruses, including human metapneumovirus (HMPV) [4], respiratory syncytial virus (RSV) [5], and sudden acute respiratory syndrome coronavirus SARS-CoV [6], as well as non-respiratory viruses such as human immunodeficiency virus (HIV) [7], hepatitis C virus (HCV) [8], Zika virus (ZIKV) [9], and Marburg and Ebola viruses [6]. 

Various techniques can be used to allow for ectopic overexpression of a particular protein within host cells. Transduction of cell lines using lentiviral expression vectors achieves integration of the introduced gene into the host genome, resulting in long-term, stable protein expression [10]. Plasmid-based transfection approaches have also been widely used, generally resulting in transient and sometimes high-level protein expression (reviewed in [11]). Additional important differences between overexpression systems include the specific promoter elements used to drive protein expression, the presence or absence of an N- or C-terminal tag on the protein of interest, the use of antibiotic resistance markers to select for stably transfected cell lines versus transient expression, and the cell type used for overexpression.

We recently utilised a doxycycline (DOX)-inducible protein overexpression system in A549 airway epithelial cells to demonstrate that IFITM1, IFITM2, and IFITM3 are cellular host factors that inhibit IAV but not parainfluenza virus (PIV)-3 infection [12]. Importantly, our study incorporated the siRNA knockdown of endogenous IFITM proteins to substantiate these findings [12]. It was intriguing that the lack of IFITM-mediated restriction towards PIV-3 in our study contradicted two previous reports using overexpression approaches to ascribe an antiviral role to IFITM1 against this virus [13,14]. 

Herein, we overexpressed each of the three IFITM proteins in A549 cells using a constitutive overexpression system. In direct contrast to our earlier study [12], we observed significant restriction of PIV-3 infection by all three IFITM proteins when constitutively overexpressed in the same cell type. Furthermore, only constitutive expression of IFITM2 and IFITM3, but not IFITM1, was associated with reduced IAV infection. Variable IFITM mRNA and protein expression levels in A549 cells with constitutive versus inducible overexpression of each IFITM were detected, and we hypothesise that this may contribute to differential antiviral activity towards different viruses in different cell types. Our findings clearly highlight the caveats associated with the overexpression approaches used to screen cellular host proteins for antiviral activity.

## 2. Materials and Methods

### 2.1. Cells

A549 human lung epithelial cells (American type culture collection (ATCC), Manassas, VA) were maintained and passaged in Kaighn’s modification of Ham’s F-12 medium (Gibco, Thermo Fisher Scientific, Whaltam, MA, USA) supplemented with 10% (*v*/*v*) foetal calf serum (FCS) (Sigma-Aldrich, St. Louis, MO, USA), 2 mM L-glutamine (Gibco), 1 mM sodium pyruvate (Gibco), 100 units/mL penicillin, and 100 µg/mL streptomycin (Gibco). Madin–Darby canine kidney (MDCK) cells (ATCC) were maintained and passaged in RPMI 1640 (Gibco) supplemented as above. LLC-MK2 monkey kidney epithelial cells (ATCC) were cultured in Opti-MEM I (Gibco) supplemented as above but with 5% (*v*/*v*) FCS. All cell lines were mycoplasma-free and tested on a regular basis with the Mycoalert mycoplasma detection kit (Lonza, Basel, Switzerland).

### 2.2. Viruses

Human parainfluenza virus (PIV)-3 strain C243 was obtained from the ATCC (VR1540) and propagated in LLC-MK2 cells. Briefly, LLC-MK2 cells were inoculated at a multiplicity of infection (MOI) of 0.1 in serum-free media for 1 h at 37 °C and then maintained in 2% (*v*/*v*) FCS until a cytopathic effect (CPE) of approximately 80% was observed (5–6 days post-infection). Supernatants were collected and clarified by centrifugation, and virus titres were determined by titration on LLC-MK2 cells followed by immunofluorescence staining using antibody directed to the PIV-3 hemagglutinin-neuraminidase (HN) protein (Abcam, Cambridge, UK) in conjunction with fluorescein isothiocyanate (FITC)-conjugated goat anti-mouse IgG (Millipore Sigma, Burlington, MA, USA). Virus titres were expressed as fluorescent-focus units (FFU)/mL.

The IAV strain A/Brazil/11/78 (Brazil 78; H1N1) was propagated in the allantoic cavity of 10-day embryonated eggs and titres of infectious virus determined by plaque assay on MDCK cells [15]. Embryonated eggs were provided by Seqirus, Parkville, Australia, and were infected with approval from the Biochemistry and Molecular Biology, Dental Science, Medicine, Microbiology and Immunology, and Surgery Animal Ethics Committee at The University of Melbourne in accordance with the NHMRC Australian code of practice for the care and use of animals for scientific purposes.

### 2.3. A549 Cells with Constitutive Overexpression of Human IFITM1, IFITM2, and IFITM3 Proteins

A549 cells with stable constitutive IFITM expression were generated using neomycin-resistant pcDNA3 plasmids encoding the same N-terminally FLAG-tagged human IFITM1, IFITM2, or IFITM3 constructs used in our previous study [12]. Briefly, the coding sequences of human IFITM1, IFITM2, and IFITM3 proteins (NCBI consensus coding sequences IFITM1: CCDS41584.1; IFITM2: CCDS41583.1; IFITM3: CCDS41585.1) containing an N-terminal FLAG tag (peptide sequence DYKDDDK) were synthesized as geneblocks (GeneArt Gene Synthesis, ThermoFisher Scientific, Carlsbad, CA, USA). A549 control cells were also engineered to express an irrelevant protein without a FLAG tag, cytoplasmic hen egg ovalbumin (OVA) (lacking the sequence for cell-surface trafficking [16], obtained from a plasmid generously donated by A. M. Lew and J. L. Brady, The Walter and Eliza Hall Institute, Parkville, Australia). Stably transfected cells were selected in the presence of 500 μg/mL geneticin (G418, Invitrogen (ThermoFisher), Carlsbad, CA, USA), followed by generation of clonal cell populations from single cells by limiting dilution as described previously [17]. A549 cell lines with constitutive IFITM control protein overexpression were maintained in media containing 1 mg/mL geneticin (G418, Invitrogen). For infection experiments, cells were seeded and incubated overnight in the absence of geneticin.

### 2.4. A549 Cells with Inducible Overexpression of Human IFITM1, IFITM2, and IFITM3 Proteins

The generation of A549 cells with DOX-inducible IFITM1, IFITM2, and IFITM3 (or control protein) expression has been described previously [12]. In order to induce the expression of IFITM proteins, A549 cells were incubated in media containing 1 µg/mL of DOX (Sigma-Aldrich) for 24 h at 37 °C. At 24 h post-DOX-induction, intracellular FLAG expression (as a measure of IFITM protein induction) was confirmed by flow cytometry.

### 2.5. RT-qPCR to Detect IFITM mRNA

Total RNA was isolated from A549 cells using the RNeasy plus mini kit (Qiagen, Germantown, MD, USA) according to manufacturer’s instructions. cDNA was prepared using the SensiFAST cDNA synthesis kit (Bioline, Memphis, TN, USA). qPCR was performed using the SensiFast 2x SYBR Lo-Rox kit (Bioline) with primer pairs specific for human IFITM1 (forward: AGCATTCGCCTACTCCGTGAAG; reverse: CACAGAGCCGAATACCAGTAACAG), human IFITM2 (forward: GAACCACATTGTGCAAACCTTCTCTC; reverse: TTCCTGCTCCTCCTTGAGCATC), or human IFITM3 (forward: ATCGTCATCCCAGTGCTGAT; reverse: ACGTGGGATACAGGTCATGG). Human GAPDH (forward: TGAAGGTCGGAGTCAACGG; reverse: GGCAACAATATCCACTTTACCAGAG), RPL13a (forward: GCCCCTGTTTCAAGGGATAAGA; reverse: CCTCGACCATCAAGCACCA), and TBP (forward: GCACTGATTTTCAGTTCTGGGA; reverse: GCTGGAAAACCCAACTTCTGT) expression was used for normalisation. Data acquisition was performed using the QuantStudio 7 Flex Real-Time PCR System and Analysis Software (Applied Biosystems (ThermoFisher), Foster City, CA, USA). Statistical analysis was performed using GraphPad Prism (GraphPad Software, San Diego, CA, USA).

### 2.6. Flow Cytometric Detection of IFITM Proteins

A549 cells expressing IFITM FLAG-tagged proteins were detached and stained with the fixable viability dye eFlour780 or eFluor450 (eBioscience (ThermoFisher), San Diego, CA, USA) before fixation with 4% (*v*/*v*) paraformaldehyde (PFA) in phosphate-buffered saline (PBS). After fixation, cells were permeabilized in PBS containing 0.5% (*v*/*v*) Triton X-100 (Sigma-Aldrich) and stained with an anti-FLAG-allophycocyanin (APC) mAb (Clone L5, Biolegend, San Diego, CA, USA). Samples were analysed on a FACSCanto II (BD Biosciences, CA, USA) or a LSRFortessa flow cytometer (BD Bioscience), before data analysis using FlowJo software version 10.6.2.

### 2.7. Western Blot Detection of IFITM Proteins

Whole cell lysates were prepared using a buffer comprising 50 mM Tris-HCL (pH 7.5), 150 mM NaCl, 0.5% (*v*/*v*) Triton X-100, 1 mM CaCl_2_, 1 mM MgCl_2_, and a broad-spectrum protease inhibitor cocktail (Roche, Manheim, Germany). Samples were heated to 90 °C for 5 min before separation by SDS-PAGE under reducing conditions using 4–15% gradient gels (Biorad), followed by transfer to a polyvinylidene difluoride (PVDF) membrane (Millipore) in Tris-glycine transfer buffer (25 mM Tris containing 192 mM glycine and 10% (*v*/*v*) methanol; pH 8.3). Membranes were blocked in PBS containing 5% (*w*/*v*) bovine serum albumin (BSA) (Sigma-Aldrich) and 0.1% (*v*/*v*) Tween-20 (Sigma-Aldrich). All subsequent wash and antibody-binding steps were performed in PBS containing 0.05% (*v*/*v*) Tween-20. Cellular β-actin (approx. 43 kDa) was monitored to ensure equivalent protein loading of all samples using a mouse monoclonal antibody to β-actin (clone sc-47778; Santa Cruz Biotechnology, Dallas, TX, USA) in conjunction with rabbit anti-mouse HRP (Dako (Agilent Technologies), Santa Clara, CA, USA). An anti-FLAG antibody (anti-FLAG (M2), Sigma-Aldrich) was used to detect IFITM proteins in conjunction with HRP-conjugated secondary antibodies against mouse or rabbit immunoglobulins (Dako) and enhanced chemiluminescence (ECL) (Amersham) according to the manufacturer’s instructions and visualised on an Amersham Imager 600 Series (GE Healthcare, Chicago, IL, USA). For the detection of IFITM proteins using IFITM-specific antibodies, anti-IFITM1 (Proteintech, Rosemont, IL, USA number 60074-1-Ig), anti-IFITM2 (Proteintech number 66137-1-Ig), and anti-IFITM3 (Cell Signaling Technology (CST), Danvers, MA, USA number 59212S) were used in conjunction secondary antibodies against mouse or rabbit immunoglobulins.

### 2.8. IFITM Protein Localisation by Fluorescence Microscopy

A549 cells were seeded into 8-well chamber slides (Nunc Lab-Tek, Millipore-Sigma, Burlington, MA, USA) and incubated overnight. Biotinylated Sambucus nigra agglutinin (SNA) lectin (Vector Laboratories, Burlingame, CA, USA) diluted in Tris-buffered saline (TBS) with 10 mM CaCl_2_ and 1% (*w*/*v*) BSA, in conjunction with streptavidin-AlexaFluor-647 (Life Technologies, ThermoFisher), were used to stain the cell surface. For intracellular staining, cell monolayers were fixed in 4% (*w*/*v*) PFA, permeabilised in PBS with 0.1% (*v*/*v*) Triton-X 100, and blocked in PBS with 5% (*w*/*v*) BSA followed by 5% (*v*/*v*) FCS. Intracellular IFITMs were detected via the FLAG tag using a mouse anti-FLAG antibody conjugated to FITC (M2 clone, Sigma-Aldrich), in conjunction with an anti-mouse AlexaFluor-488 antibody (Life Technologies) to enhance the FLAG signal. Early endosomes, late endosomes, and lysosomes were stained with an anti-EEA1 antibody (Abcam; #2900), anti-Rab7 antibody (Abcam; #137029) or anti-LAMP1 antibody (Abcam; #24170), respectively, in conjunction with the secondary donkey anti-rabbit AlexaFluor-647 (Life Technologies). Nuclei were counterstained with DAPI before mounting with ProLong Gold (Life Technologies). Images were acquired on a Zeiss LSM 780 laser scanning microscope and analysed with ImageJ software (Fiji distribution [18]). A correlation analysis between IFITM protein expression (FLAG signal) and cellular membranes (surface or endosomal marker expression) was performed. Pearson’s coefficient (R) was calculated with the JACoP plugin [19] as an indicator of co-localisation. 

### 2.9. Virus Infection Assays 

Cell monolayers in 24-well tissue culture plates (Corning Incorporated, Corning, NY, USA) were infected with IAV or PIV-3 (at the MOIs indicated) in serum-free media for 1 h at 37 °C in the presence of 5% CO_2_. The viral inoculum was removed before cell monolayers were washed thoroughly to remove residual input virus and then maintained in serum-free media at 37 °C in 5% CO_2_. Viral infection was quantitated by intracellular staining of newly synthesised viral proteins in conjunction with flow cytometric analysis. Newly synthesised IAV nucleoprotein (NP) was detected 8 hours post-infection (hpi) using an anti-IAV NP antibody (mAb MP3.10g2.1C7, from the WHO Collaborating Centre for Reference and Research on Influenza, Melbourne, Australia) in conjunction with a FITC-conjugated goat anti-mouse IgG (Millipore) [20]. Newly synthesised PIV-3 HN protein was detected 18 hpi as described previously [21] using an anti-PIV-3 HN antibody (Abcam) in conjunction with a FITC-conjugated goat anti-mouse IgG (Millipore). 

### 2.10. Virus Binding Assay

In order to evaluate virus binding to cell lines expressing different IFITM proteins, we used highly purified virus preparations in combination with flow cytometric detection of cell-surface bound virus particles. LLC-MK2 cells were infected with PIV-3 and cell supernatants were concentrated by ultracentrifugation through a sucrose cushion, and then pelleted by ultracentrifugation. IAV grown in the allantoic cavity of 10-day embryonated eggs was purified by precipitation with polyethylene glycol (PEG) followed by ultracentrifugation using a sucrose gradient and concentrated with an additional centrifugation step. The protein concentration of each virus was determined by Bradford protein assay (Bio-rad), according to the manufacturer’s instructions. For analysis of PIV-3 and IAV binding to the cell surface by flow cytometry, cells were detached and washed with cold lectin buffer (TBS with 10 mM CaCl_2_ and 1% (wt/vol) BSA) and all subsequent steps were performed on ice. Detached cells were incubated with 5 μg/mL of purified IAV (BJx109, a high-yielding reassortant of A/PR/8/34 (PR8, H1N1) and A/Beijing/353/89 (BJ89, H3N2)) or 10 μg/mL purified PIV-3 in lectin buffer for 30 min. After washing, bound IAV was detected with 5 μg/mL of biotinylated MAb C1/1 recognizing the HA of BJ89 (kindly provided by Professor Lorena Brown, The University of Melbourne), in conjunction with streptavidin-APC (Invitrogen). Bound PIV-3 was detected using an anti-PIV-3 HN antibody (Abcam) in conjunction with a FITC-conjugated goat anti-mouse IgG (Millipore).

### 2.11. Statistical Analysis

Graphing and statistical analysis of data were performed using GraphPad Prism (GraphPad Software, San Diego, CA, USA). P-values were calculated by one-way analysis of variance (ANOVA) followed by post-hoc analysis using Tukey’s multiple comparative test or an unpaired two-tailed *t*-test as indicated.

## 3. Results

### 3.1. Generation of A549 Cells with Stable Constitutive Overexpression of IFITM Proteins

**Figure 1:**
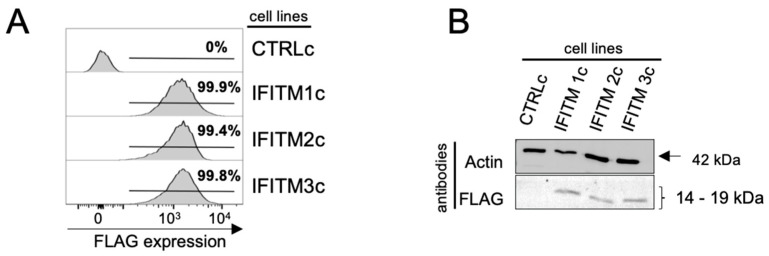
Constitutive overexpression of IFITM1, IFITM2, and IFITM3 in A549 cells. A549 cells with stable constitutive IFITM overexpression (IFITMc) were generated by transfection of pcDNA3 plasmids expressing IFITM1, IFITM2, or IFITM3 constructs with an N-terminal FLAG-tag [12] or a control protein (cytoplasmic ovalbumin (OVA) [16]; CTRLc), and clonal populations were established as described previously [17]. (**A**) A549 IFITM1c, IFITM2c, IFITM3c, or CTRLc cells were assessed by intracellular staining for FLAG protein in conjunction with flow cytometric analysis. Representative histograms are shown. (**B**) Lysates from A549 IFITM1c, IFITM2c, IFITM3c, or CTRLc cells were prepared, resolved by SDS-PAGE under reducing conditions, and transferred to a polyvinylidene fluoride (PVDF) membrane. Proteins were detected by Western blot, using an anti-FLAG antibody directly conjugated to APC. Beta-actin (42kDa) expression was monitored as a loading control.

We used a pcDNA3 plasmid transfection approach and generated clonal A549 cell lines with constitutive (c) overexpression of the same IFITM1 (IFITM1c), IFITM2 (IFITM2c), or IFITM3 (IFITM3c) constructs used previously in our inducible overexpression study (i.e., N-terminally FLAG-tagged IFITM proteins and an untagged cytoplasmic ovalbumin as control (CTRLc)) [12]. IFITM protein overexpression in each respective A549 cell line was determined by detecting intracellular FLAG using flow cytometry, where 99.9% (IFITM1c), 99.4% (IFITM2c), and 99.8% (IFITM3c) of viable cells were FLAG-positive compared with the control cell line (CTRLc) (Figure 1A). IFITM proteins were also detected via the FLAG tag using Western blot analysis of A549 cell lysates, with bands detected at the expected size for each respective IFITM protein (Figure 1B). Non-cross-reactive IFITM1, IFITM2, and IFITM3-specific antibodies [12,22] were also used to validate the specificity of each respective IFITM protein by Western blot analysis (Supplementary Figure S1).

### 3.2. Assessing Antiviral Activity against IAV and PIV-3 in A549 Cell Lines That Constitutively Overexpress Different IFITM Proteins

**Figure 2:**
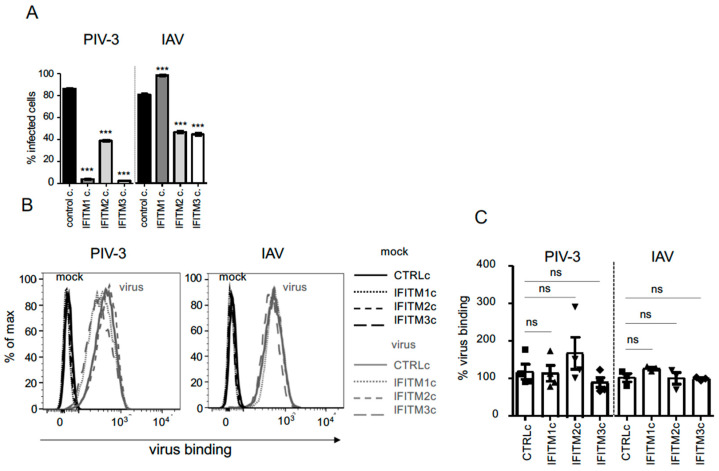
Assessing the antiviral activity of IFITM1, IFITM2, and IFITM3 against PIV-3 and IAV following constitutive overexpression in A549 cells. (**A**) A549 cell lines with stable constitutive overexpression of IFITM1, IFITM2, and IFITM3 (IFITMc), or control protein (CTRLc) were infected with PIV-3 (strain C243; multiplicity of infection (MOI) = 2) or IAV (A/Brazil/11/78 (Brazil 78; H1N1); MOI = 5) for 18 h (PIV-3) or 8 h (IAV). Infection was determined by staining the PIV-3 hemagglutinin-neuraminidase (HN) protein using an anti-HN antibody (Abcam, UK) or IAV nucleoprotein (NP) using an anti-NP monoclonal antibody (MP3.10g2.1C7) in conjunction with flow cytometry [12]. Data are representative of three independent experiments performed in triplicate. Error bars are the standard error of the mean (SEM). *** *p* ≤ 0.001 (one-way analysis of variance (ANOVA) with Tukey’s multiple comparative analysis). (**B**,**C**) Purified PIV-3 (10 ug/mL) and purified IAV (5 ug/mL) bound to A549 cells with constitutive overexpression of IFITM1, IFITM2, and IFITM3 (IFITMc), or control protein (CTRLc) were detected using an anti-PIV-3 HN antibody in conjunction with a FITC-conjugated goat anti-mouse IgG or an anti-IAV HA antibody in conjunction with a streptavidin-APC conjugate and flow cytometry. (**B**) Representative histogram overlays of a minimum of 3 independent experiments of mock (black)- and virus-exposed (gray) CTRLc and IFITMc cell lines. Fluorescence intensity represents the amount of virus bound to the cell surface (virus binding). (**C**) The binding of virus cells is represented as the geometric mean fluorescence (gMFI) relative to the parental A549 cells (100% virus binding). Each data point represents an individual experiment (n = 4 for PIV-3; n = 3 for IAV). Error bars are SEM, significance by one-way analysis of variance (ANOVA) with Tukey’s multiple comparative analysis, *** *p* ≤ 0.001, ns *p* > 0.05 for (**A**,**C**).

We utilised the constitutively overexpressing IFITM1c, IFITM2c, and IFITM3c A549 cell lines to assess the antiviral activity of each IFITM protein against the early stages of IAV and PIV-3 infection. Following infection, a significant decrease in the percentage of PIV-3-infected cells was detected at 18 h post-infection (assessed by intracellular staining for the viral hemagglutinin neuraminidase (HN) protein) in cell lines with constitutive overexpression of each IFITM protein compared with control cells (Figure 2A). For IAV, the overexpression of IFITM2 and IFITM3 was associated with a significant reduction in infected cells at 8 h post-infection (assessed by intracellular staining for the viral nucleoprotein (NP) protein, (Figure 2A)). Surprisingly, the percentage of IAV-infected cells was not reduced in IFITM1c cells but was significantly enhanced compared with control cells (Figure 2A). The significant restriction of PIV-3 infection by IFITM1c, IFITM2c, and IFITM3c in A549 cells is in direct contrast with our prior published observations using inducible overexpression of the same IFITM constructs in the same cell type [12]. While IFITM2 and IFITM3 restriction of IAV infection is in agreement with our findings using the inducible system, enhanced IAV infection following constitutive IFITM1 overexpression is not [12], nor is it consistent with the findings of others [3,6]. A significant restriction of PIV-3 infection by IFITM1c, IFITM2c, and IFITM3c was still observed using a lower MOI (MO1 = 0.1) (data not shown). Similarly, the same pattern of restriction/enhancement was also detected using a low MOI for IAV infection (MOI = 1) of IFITM1c, IFITM2c, and IFITM3c cells (data not shown). In order to determine whether the increased susceptibility of IFITM1c cells to IAV infection was due to enhanced virus binding to the cell surface, we performed a virus binding assay. Similar amounts of IAV bound to each cell line, and there were no significant differences between the control cells (CTRLc) and any of the cells that constitutively overexpressed IFITM proteins, including IFITM1c (Figure 2B,C). Equivalent amounts of PIV-3 were also observed bound to each cell line with constitutive IFITM (or control) overexpression (Figure 2B,C). 

### 3.3. Comparison of IFITM1, IFITM2, and IFITM3 mRNA and Protein Levels in Constitutive versus Inducible Overexpressing A549 Cell Lines

**Figure 3:**
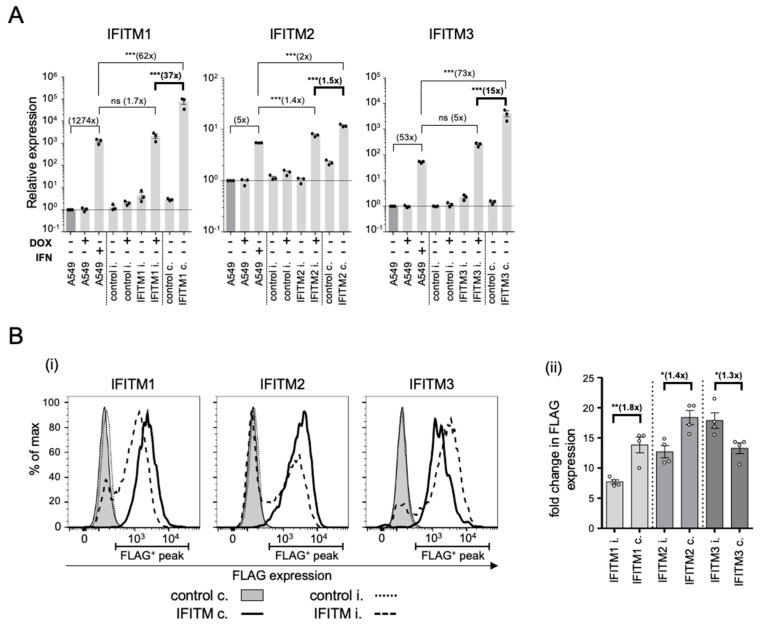
IFITM expression in A549 cells with constitutive and inducible overexpression. (**A**) IFITM mRNA levels in A549 cell lines with constitutive (IFITMc) IFITM expression or in A549 cell lines with inducible (IFITMi) IFITM overexpression (generated previously by selecting for cells with high inducible IFITM expression [12]) were quantitated 24 h after treatment with doxycycline (DOX), human interferon-alpha (IFN) (Lonza), or mock treatment as described before [12]. IFITM mRNA expression was normalised to three housekeeping genes (GAPDH, RPL13a, TBP) and is presented as a comparison with mock-treated A549 cells (relative expression). Data are representative of two independent experiments performed in triplicate. *** *p* ≤ 0.001, ns *p* > 0.05 (one-way analysis of variance (ANOVA) with Tukey’s multiple comparative analysis). (**B**) IFITM protein levels in A549 cells with constitutive (IFITMc) or inducible (IFITMi) expression (24 h post DOX-induction) were quantitated by intracellular FLAG staining and flow cytometry. (**i**) Representative histograms of IFITM1, IFITM2, and IFITM3 protein expression. (**ii**) Fold-change in IFITM protein expression compared with control cells (geometrical mean fluorescence intensity (gMFI) of the FLAG-positive peak (as indicated in (**i**)) divided by the gMFI of control cells). Data are pooled from 4 experiments performed in triplicate (** *p* ≤ 0.01, * *p* ≤ 0.05, unpaired, two-tailed *t*-test).

Given the unexpected differences in antiviral activity of the IFITM proteins against IAV or PIV-3 using constitutive or inducible protein overexpression, we next compared IFITM mRNA and protein expression levels in each cell line. We measured mRNA levels using IFITM-specific primers to detect both endogenous and overexpressed transcripts. The analysis showed significantly higher levels of IFITM1 (37x) and IFITM3 (15x) mRNA in the constitutively overexpressing cell lines compared with the inducible cell lines (Figure 3A). Induced IFITM1 and IFITM3 mRNA levels were comparable with the expression of endogenous genes in IFN-treated A549 cells (Figure 3A). IFITM2 mRNA levels were more consistent across the two overexpressing cell lines, where a 1.4-fold and a 2-fold increase in the level of IFITM2 mRNA (compared to IFN-treated A549 cells) was detected in constitutive and inducible overexpressing cells, respectively (Figure 3A). 

In order to determine whether high levels of IFITM mRNA in the constitutively expressing cell lines correlate with increased protein abundance, we compared the expression of FLAG-tagged IFITM proteins in the inducible and constitutive overexpressing cell lines using flow cytometry. More IFITM1 (1.8×) and IFITM2 (1.4×) proteins were detected in cells with constitutive rather than induced overexpression (Figure 3B,C). For IFITM3 however, inducible overexpression led to higher levels of protein (1.3x) when compared to constitutive overexpression (Figure 3B,C).

**Figure 4:**
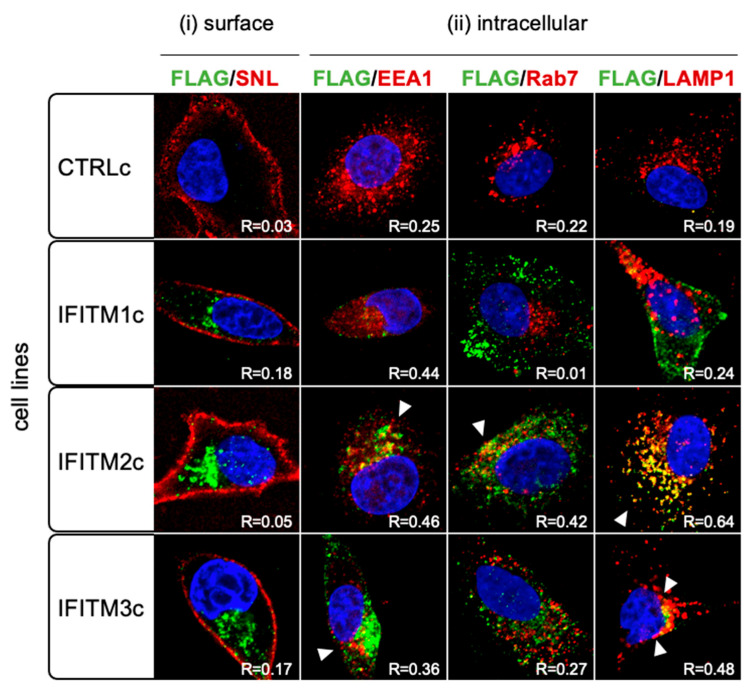
IFITM protein localization in A549 cells with constitutive IFITM overexpression. IFITM1c, IFITM2c, or IFITM3c, or CTRLc A549 cells were (**i**) surface-stained with biotinylated SNL (in conjunction with a streptavidin-Texas Red conjugate) then intracellularly stained with a FITC-conjugated anti-FLAG antibody (M2 clone) in conjunction with an anti-mouse AlexaFluor-488 to enhance fluorescence (green) or (**ii**) stained for intracellular FLAG (green) followed by staining with antibodies against EEA1, Rab7, or LAMP1 (each in conjunction with an anti-rabbit AlexaFluor-568) (red)). Cells were then mounted with Vectashield Antifade Mounting Medium with DAPI (Vector Labs) and imaged by confocal microscopy. Pearson’s coefficient (R) was calculated between the green and red channels, indicating the degree of co-localization (orange; white arrows). Magnification was 63x. Images are representative of those acquired from at least three independent experiments.

We previously showed in A549 cells that the distribution of inducible IFITM1, IFITM2, and IFITM3 overexpressed proteins was consistent with the expected cellular localisation of endogenous protein [12]. To investigate the cellular localisation of constitutively overexpressed IFITM proteins, cells were stained with an anti-FLAG antibody (green) and visualised in conjunction with cell-surface sialic acid (detected using Sambucus nigra lectin (SNL; red)) and intracellular endosomal compartments (stained using antibodies against EEA1, Rab7, or LAMP1 (red)) (Figure 4). We found that IFITM1c did not strongly co-localise with either the cell surface (as indicated by a low Pearson correlation coefficient (R) of 0.18 between the SNL and FLAG staining; Figure 4) or intracellular endosomal compartments (R < 0.5 for EEA1/FLAG, Rab7/FLAG, or LAMP1/FLAG), while IFITM2c strongly co-localized with lysosomes (R = 0.64 for LAMP1/FLAG) and had a weak positive correlation with EEA1 and Rab7 staining (R= 0.46 and 0.42, respectively). There was no appreciable co-localization of IFITM2c with surface SNL (R = 0.05). IFITM3c did not co-localise with the cell surface (R = 0.17 for SNL/FLAG), and there was little evidence of co-localization with EEA1 (R = 0.36) or Rab7 (R = 0.27), but there was some possible lysosomal distribution of IFITM3c (R = 0.48 for LAMP1/FLAG). These data suggest that the localisation of each overexpressed IFITM protein detected via the FLAG tag was comparable between the constitutive and inducible systems in A549 cells. 

## 4. Discussion

The potential antiviral activity of host proteins is often evaluated by ectopic overexpression of the respective protein in immortalised cell lines. Here, we show that the generation of A549 cell lines with constitutive and inducible overexpression of the same IFITM constructs using two different approaches resulted in (i) variable levels of IFITM mRNA and protein and (ii) variable antiviral activity against two respiratory viruses, IAV and PIV-3. It is becoming increasingly clear that variations in IFITM expression levels can directly impact their antiviral activity against different viruses in different cell types. In cell culture, 293T cells transfected with increasing amounts of IFITM1, IFITM2, or IFITM3 expression plasmids showed a dose-dependent increase in the expression of IFITM protein, which also correlated with the degree of inhibition of infection by a HCoV-229E-spike protein pseudotyped virus particle [23]. Similarly, research by Chesarino et al. [24] showed that disruption of the NEDD4-mediated ubiquitination of IFITM3 resulted in increased cellular IFITM3 protein levels, concurrent with enhanced protection against IAV infection of human lung cells. Doxycycline-inducible IFITM3 expression in A549 cells has also been associated with a dose-dependent increase in IAV restriction concomitant with increasing IFITM3 protein levels [25]. Herein, we show that constitutive overexpression resulted in markedly higher levels of IFITM mRNA expression, especially IFITM1 and IFITM3, compared with both the inducible system and with IFN-stimulated parental cells. This also correlated with antiviral activity against PIV-3. These results suggest that the expression of IFITM1, IFITM2, and IFITM3 well above endogenous levels, including those following IFN stimulation, may be necessary to achieve restriction of PIV-3 infection. Similar findings have been observed for ZIKV, where replication was only restricted by IFITM3 when it was overexpressed at levels higher than those induced by IFN treatment of A549 cells [26], although a role for endogenous IFITM3 in the restriction of ZIKV has also been demonstrated [27].

IFITM family members inhibit different viruses with varying efficiency, which has been attributed, at least in part, to the distinct cellular localization of IFITM1, IFITM2, and IFITM3 proteins [4,28,29,30]. In most cell types, including A549 cells, the consensus is that IFITM1 is predominantly localized to the plasma membrane [7,8,13,31], while IFITM2 and IFITM3 proteins redistribute to Rab7, CD63, and LAMP1-positive compartments of the endolysosomal system [8,32,33,34]. The link between the localization of IFITM3 to the endocytic compartment and efficient restriction of IAV replication (cell entry via endocytosis) is evidenced by experiments that showed a decrease in anti-IAV activity when IFITM3 expression was re-localised from endosomes to the cell surface [35]. However, this re-localisation of IFITM3 also resulted in enhanced inhibition of HIV-1, which enters cells via fusion at the plasma membrane [35]. Previous studies by Rabbani et al. have shown that re-localisation of IFITM3 to the cell surface in HEK293 cells, by mutating a specific site in the N-terminal domain (Y20), enhances the restriction of PIV-3 infection (entry via fusion at the plasma membrane) [14]. Given that excessive levels of exogenously expressed proteins can result in mislocalisation [36], we considered that the high levels of IFITM proteins expressed in our constitutive system (73x higher IFITM3 mRNA levels compared with IFN-α-treated parental cells, as shown in Figure 3) may cause aberrant protein localisation outside the endosomal compartment. This prompted us to investigate differences in the localisation of each IFITM protein in A549 cells following inducible versus constitutive overexpression. Although we observed only subtle differences in the expression of IFITM2 and IFITM3 within the intracellular endolysosomal compartment, it is still possible that undetected mislocalised overexpressed IFITM proteins at the plasma membrane, the site of viral entry for PIV-3, contribute to potent antiviral activity against PIV-3. 

Our prior studies using inducible IFITM1 overexpression and siRNA knockdown [12] indicated a role for IFITM1 in the restriction of IAV infection. However, constitutive overexpression of IFITM1 in A549 cells resulted in enhanced levels of IAV infection that were not due to increased virus binding to the cell surface (Figure 2B). Interestingly, Smith et al. have also reported a slight but significant enhancement of IAV A/PR/8/34 (H1N1) infection in Vero cells transduced to stably overexpress IFITM2 [13]. It is possible that long-term constitutive overexpression of IFITMs may modulate the expression and/or activity of other endogenous host proteins that influence viral replication. Two recent studies suggest that mislocalised IFITM2 [37] and IFITM3 [38] promoted infection with SARS-CoV-2. Collectively, these seemingly contradictory findings, including our own observations with IFITM1, emphasise that robust validation through complementary approaches to modulate endogenous protein expression or to mutate specific functional IFITM domains are essential to define the precise role of IFITM proteins in regulating viral infection. 

We conclude that overexpression systems are highly useful in the initial screening of cellular factors for antiviral activity. However, experimental data generated using cell lines with exceedingly high levels of overexpressed mRNA and/or protein should be interpreted with caution. This is particularly relevant for host factors where the level of expression can dictate cellular localisation patterns that may also be associated with the modulation of the architecture of distinct cellular compartments. Collectively, these factors could be anticipated to influence the degree of antiviral activity for specific viruses.

## Data Availability

Data is contained within the article or Supplementary Material.

## Supplementary Materials

The following supporting information can be downloaded at: https://www.mdpi.com/article/10.3390/pathogens12040519/s1, Figure S1: Constitutive overexpression of IFITM1, IFITM2 and IFITM3 in A549 cells detected with IFITM specific antibodies.
