# Supplementary material for: Caveats of Using Overexpression Approaches to Screen Cellular Host IFITM Proteins for Antiviral Activity
Source: Pathogens. 2023 Mar 27;12(4):519. doi: 10.3390/pathogens12040519 (PMC10145288; doi:10.3390/pathogens12040519)
Supplement: Supplementary file 1 [file pathogens-12-00519-s001.zip › pathogens-2228601-supplementary.pdf]

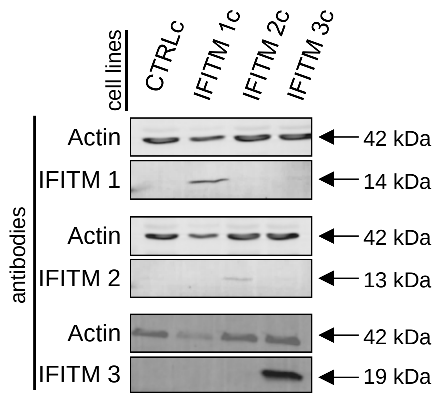

Supplementary Figure 1: Constitutive overexpression of IFITM1, IFITM2 and IFITM3 in A549 cells detected with IFITM specific antibodies.

Lysates from A549 IFITM1c, IFITM2c, IFITM3c or CTRLc cells were prepared, resolved by SDS-PAGE under reducing conditions and transferred to a polyvinylidene fluoride (PVDF) membrane. Proteins were detected by western blot, using IFITM-specific antibodies in conjunction with anti-mouse AlexaFluor-488 (IFITM1c and IFITM2c) or anti-rabbit AlexaFluor-568 (IFITM3c). Beta-actin (42kDa) expression was monitored to ensure equivalent loading between samples.
